# lncRNA *H19* acts as a ceRNA to regulate the expression of *CTGF* by targeting miR-*19b* in polycystic ovary syndrome

**DOI:** 10.1590/1414-431X20209266

**Published:** 2020-10-07

**Authors:** Xiuhong Sun, Xiumin Yan, Kailiang Liu, Min Wu, Zhongyi Li, Yao Wang, Xingming Zhong, Li Qin, Chuican Huang, Xiangcai Wei

**Affiliations:** 1 School of Medicine, Jinan University, Guangzhou, Guangdong Province, China; 2 Guangdong Women and Children Hospital, Guangzhou, Guangdong Province, China

**Keywords:** Polycystic ovary syndrome, Long non-coding RNA *H19*, microRNA-*19b*, CTGF

## Abstract

The etiology of polycystic ovary syndrome (PCOS) is complex and the pathogenesis is not fully understood. Some studies have shown that dysregulation of ovarian granulosa cells may be related to abnormal follicles and excessive androgen in women with PCOS. Our team has also confirmed the high expression status of *H19* in PCOS patients in the early stage. However, the relationship between *H19* and miR-*19b* in the development of PCOS is still unknown. Therefore, we used bioinformatics to predict the binding sites of human *H19* and miR-*19b*, and of miR-*19b* and *CTGF* genes. After the silencing and overexpression of *H19*, real-time polymerase chain reaction (PCR) was used to detect the expressions of *H19*, miR-*19b*, and *CTGF*. Western blotting was used to detect CTGF protein. Proliferation of KGN cells after *H19* silencing was detected by CCK8. Flow cytometry was used to detect the apoptosis of KGN cells after *H19* silencing. After the overexpression of *H19*, it was found that the expression of miR-*19b* gene decreased and the expression of *CTGF* increased, whereas silencing of *H19* did the opposite. In addition, *H19* could promote cell proliferation and decrease cell apoptosis. Finally, luciferase reporter assays showed that the 3′-end sequences of lncRNA *H19* and *CTGF* contained the binding site of miR-*19b*. In conclusion, our study indicated that lncRNA *H19* acted as a ceRNA to bind to miR-*19b* via a “sponge” to regulate the effect of *CTGF* on KGN cells, which may play a vital role in PCOS.

## Introduction

Polycystic ovary syndrome (PCOS) is a highly heterogeneous and complex disease that is one of the most common reproductive endocrine diseases in gynecology (1). It affects both birth and metabolism. Its incidence is rising, and the number of patients is large, reaching 5–10% of women of childbearing age (2). The etiology and pathogenesis of PCOS have become hotspots in many disciplines such as obstetrics and gynecology, endocrinology, and reproductive science. Although the exact cause of PCOS is still not fully understood, the survival and proliferation of granulosa cells may be the cause of PCOS (3). There is evidence that dysfunction of granulosa cells may lead to the formation of abnormal follicles in PCOS patients, but the specific mechanism remains to be determined. For PCOS, a large number of studies have confirmed that *CTGF* is highly expressed in the ovaries of PCOS patients and PCOS animal models (4). Experiments have confirmed that *CTGF* is involved in PCOS granulosa cell proliferation, and high expression of *CTGF* promotes granulosa cell proliferation and inhibits apoptosis (5).

Noncoding RNA (ncRNA) is an RNA that does not encode a protein in the body. In the last decade, with the development of sequencing technology, most RNA transcripts do not encode proteins. Depending on the number of RNAs, it can be divided into short non-coding RNAs (such as miRNAs) and long non-coding RNAs (such as lncRNA, circRNA, etc.) (6). MicroRNAs (miRNAs) are a class of highly conserved, non-coding, short-stranded RNAs that have approximately 20–23 nucleotides in length. miRNAs regulate post-transcriptionality by base pairing with the 3′ untranslated region (UTR) of the target mRNA (7). Horizontal mRNA transcription and translation affect biological function. lncRNAs are a class of RNAs that are more than 200 nucleotides in length and have limited protein-coding potential (8). Studies have shown that lncRNAs have many functions in various pathophysiological processes. The dysfunction of IncRNAs plays a key role in many diseases. The function of lncRNA is complex. In 2011, Karreth et al. (9) proposed a theory called competitive endogenous RNA (ceRNA) hypothesis that lncRNAs, mRNAs, and pseudogenes can competitively bind to microRNA response elements (MRE) and lead to mutual regulation of expression, which provides a new gene regulation mechanism.

In recent years, in order to more deeply explore the etiology of PCOS from the epigenetics, researchers are beginning to pay attention to the relationship between non-coding RNAs and PCOS. Huang et al. (10) reported that lncRNA *H19* competes with STAT3 for binding to miR-*19*, which in turn affects STAT3 expression. Zhong et al. (11) found that in PCOS, miR-*19b* is lowly expressed in ovarian tissue and granulosa cells, and miR-*19b* can regulate the proliferation of granulosa cells. However, there are no reports on the interaction between *H19* and miR-*19b* in PCOS. Therefore, this study focused on the relationship between *H19* and miR-*19b* in KGN cells, its effect on gene *CTGF*, and explored the role of *H19* in the development of PCOS.

## Material and Methods

### Cell recovery and culture

A thermostatic water bath was preheated to 37°C, and the table top was wiped and ultra-cleaned with 75% ethanol beforehand. Then, an ultraviolet light was turned on to illuminate the table for 30 min. The cryotube was quickly placed into a 37°C constant temperature water bath, and shaken continuously, so that the cell cryopreservation medium in the cryotube was melted rapidly and taken out. The tube was centrifuged at 800 *g* for 5 min at 4°C; the supernatant was discarded, 1-mL of medium was added, and the cells were shaken as appropriate to resuspend the cells. The cells were then placed in culture dishes, which were placed in a carbon dioxide incubator at 37°C for constant temperature culture for 48 h, and passed 2-3 generations to prove that the cells were viable. The culture solution was continuously changed according to the growth of the cells. The cells were rinsed in the cell culture flask 1-2 times with PBS water. A 1.5-mL trypsin-EDTA solution was added to the flask to lightly wash the cell culture dish. The trypsin-EDTA solution was discarded and the cells digested in a 37°C carbon dioxide incubator for 3 min. If cells were found to be in a circular state, 1-mL of medium was added for further digestion. The cell solution was repeatedly pipetted with a 1-mL pipette to mix the cells, and the culture solution of each flask was replenished, transferred to a new flask, and the culture solution was added in a diluted ratio.

### Real-time PCR

One millileter of Trizol was added to the cells, and the tube was shaken and mixed at 4°C for 5 min. Chloroform (0.2 mL) was added, the tube was vigorously shaken for 15 s, and then allowed to stand at 4°C for 3 min. An equal volume of isopropanol was added, mixed, and left to stand at -20°C for 20 min; the tube was then centrifuged at 12,000 *g* for 15 min at 4°C, and the supernatant removed. Centrifugation was repeated at 8000 *g* for 5 min at 4°C, and 1.5 μL of the sample solution was measured for concentration in an ultra-micro UV analyzer. Reverse transcription was performed as described in the Bestar^TM^ qPCR RT Kit (DBI, Germany).

### Cell transfection

KGN cells (80%) were removed and the old medium was aspirated. The cells were washed one or two times with PBS, trypsin solution was added, and then aspirated when the cells were separated and round pellets appeared. Fresh complete medium was added, and the cells were pipetted well to prepare a single-cell suspension. The cell density was adjusted to 8×10^4^/mL and 0.25 μL of siRNA or 0.2 μg of plasmid was added to 50 μL of serum-free medium, gently mixed, and allowed to stand at room temperature for 5 min. Lipofectamine^TM^ 2000 (0.25 μL; Thermo Fisher Scientific, USA) was added to 50 μL of serum-free medium, gently mixed, and allowed to stand at room temperature for 5 min. The diluted siRNA or plasmid was mixed with Lipofectamine^TM^ 2000, gently mixed, and allowed to stand at room temperature for 20 min to form a plasmid/Lipofectamine^TM^ 2000 complex. siRNA (100 μL) or plasmid/Lipofectamine^TM^ 2000 complex was added to the wells of the corresponding group of culture plates at a final concentration of 50 nM; after 6 h, the cells were replaced with complete medium for 48 h. The samples were subjected to qPCR and western blot detection to screen for optimal sequences.

### Cell proliferation

Cell viability was determined using Cell Counting Kit-8 (CCK8) (Dojindo, Japan) according to the instructions of the manufacturer. In brief, KGN cells during logarithmic growth period were seeded into 96-well microtiter plates at a density of 5×10^3^ cells/well. Then, cells were transfected with indicated plasmids or oligonucleotides. After transfection for 48 h, 10 μL of CCK-8 reagent was added to each well and incubated for another 4 h at 37°C and 5% CO_2_. Absorbance at 450 nm was measured to assess relative cell viability using a microplate reader (Bio-Rad, USA).

### Apoptosis

Cell apoptosis was examined using an Annexin V-FITC apoptosis detection kit (Abcam, UK) following the manufacturer's instructions. KGN cells at a density of 1×10^5^ cells/well were rinsed twice with cold PBS solution and resuspended in 1× binding buffer. Afterwards, 5 μL of Annexin V-FITC and 10 μL of propidium iodide (PI) were introduced and incubated for 10 min in the dark. Finally, apoptotic cells were determined using a flow cytometer (BD Biosciences, USA)

### Luciferase reporter assay

Bioinformatics analysis using Starbase online software (Sun Yat-sen University, China) first proved the existence of putative binding sites between *H19* and miR-*19b*. For luciferase assay, partial fragments of *H19* containing the assumed miR-*19b* binding sites were amplified and sub-cloned into psiCHECK-2 vector (Promega, USA) to generate wild-type H19 plasmid (H19-WT). Then, a Site-Directed Mutagenesis Kit (Thermo Fisher Scientific) was used to generate mutated H19 plasmid (H19-MUT). H19-WT and H19-MUT constructs were co-transfected into KGN cells along with miR-*19b* mimics or miR-NC (negative control). About 48-h post-transfection, relative luciferase activity was determined using the dual-luciferase reporter assay system (Promega).

### Statistical analysis

All experiments were performed three times. Statistical analysis was conducted using SPSS 20.0 (IBM Corp., USA), and results are reported as means±SD. Student's *t*-test or one-way analysis of variance was carried out to estimate significant group differences. P<0.05 represented a statistically significant result.

## Results

### H19 overexpression recombinant plasmid and H19 silencing site

**Figure 1:**
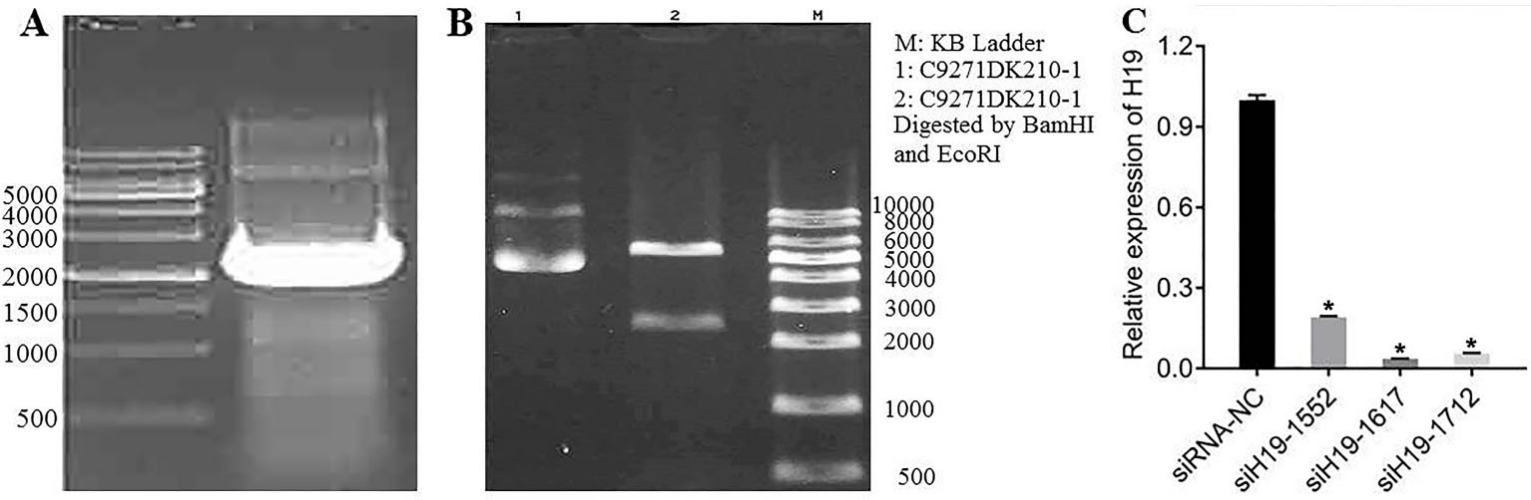
**A**, H19 PCR amplification electropherogram. **B**, Restriction endonuclease digestion of recombinant vector. **C**, Changes in H19 expression after silencing H19. Data are reported as means±SD. *P<0.05 compared to negative control (NC) (one-way analysis of variance).

Total RNA was extracted from KGN cells, and then reversed transcribed into cDNA. PCR amplified H19 by designed primers, and the product was electrophoresed, and the amplified product was 2362 bp (Figure 1A). H19 was connected to pcDNA3.0+ vector to construct H19-pcDNA 3.0+ recombinant vector and transfer it to DH5α competent cells. Monoclonal colonies were selected and restriction enzymes were used to digest the recombinant vector for PCR identification (Figure 1B).

Three silent sites, 1552, 1617, and 1712, were selected to silence H19, and the corresponding siRNA was designed and transfected into KGN cells. Compared with the control group, the expression of H19 was significantly decreased after silencing of H19 by *siH19-1552*, *siH19-1617*, and *siH19-1712* (P<0.05). Among the three silent sites, the expression of *siH19-1617* was significantly more pronounced after silencing H19, and the silencing effect was the best of the three (Figure 1C).

### Overexpression of lncRNA *H19* in KGN cells promoted the expression of *CTGF*

After transfecting the constructed H19 overexpression plasmid into KGN cells, *H19*, miR-*19b*, and *CTGF* were detected at the RNA level and protein expression by qRT-PCR and Western blot in the presence of H19 overexpression.

**Figure 2:**
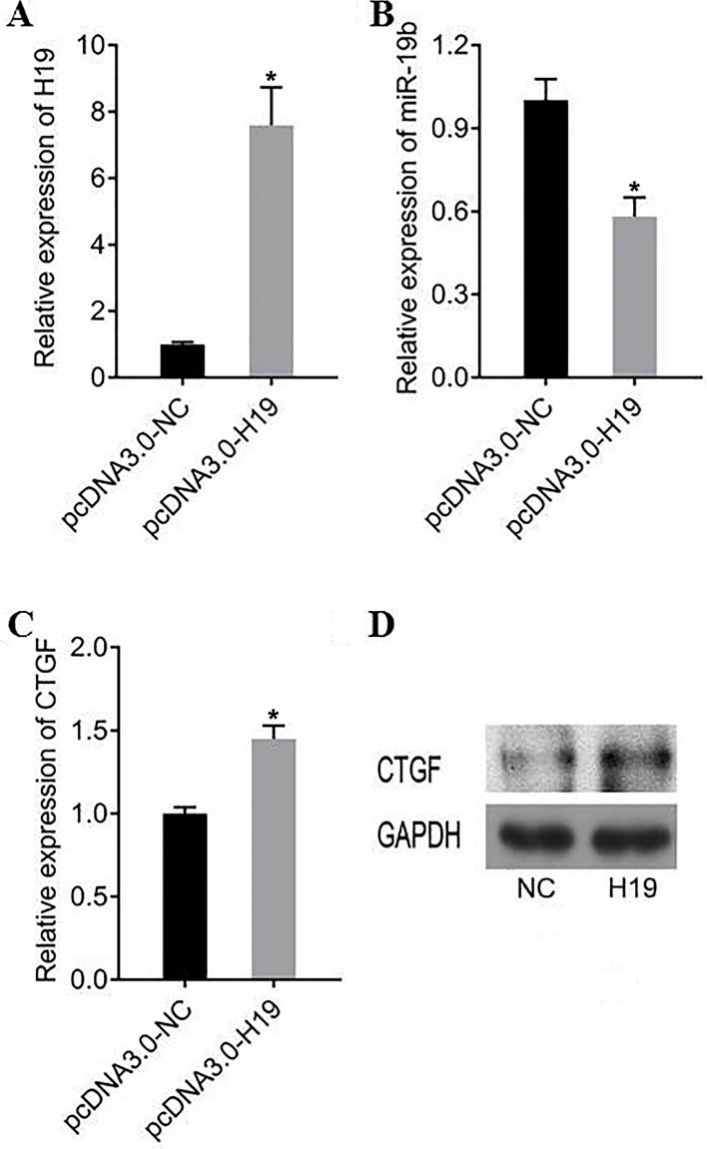
A, *H19* expression after overexpression of *H19*. **B**, Expression of miRN-*19b* after overexpression of *H19*. **C** and **D**, *CTGF* expression after overexpression of *H19*. Data are reported as means±SD. *P<0.05 compared to negative control (NC) (*t*-test).

Compared with the pcDNA3.0+NC group, the expression of *H19* transfected with the H19 overexpression plasmid group was significantly increased (P<0.05) (Figure 2A). Compared with the pcDNA3.0+NC group, miR-*19b* transfected with the H19 overexpression plasmid group was significantly decreased (P<0.05) (Figure 2B). Compared with the pcDNA3.0+NC group, *CTGF* transfected with the H19 overexpression plasmid group was significantly increased (P<0.05) (Figure 2C). The expression of CTGF protein was consistent with the expression of *CTGF* at the RNA level. After H19 overexpression, the expression of CTGF protein was also significantly increased (Figure 2D).

### Overexpression of *H19* promoted KGN cell proliferation and inhibited KGN cell apoptosis

**Figure 3:**
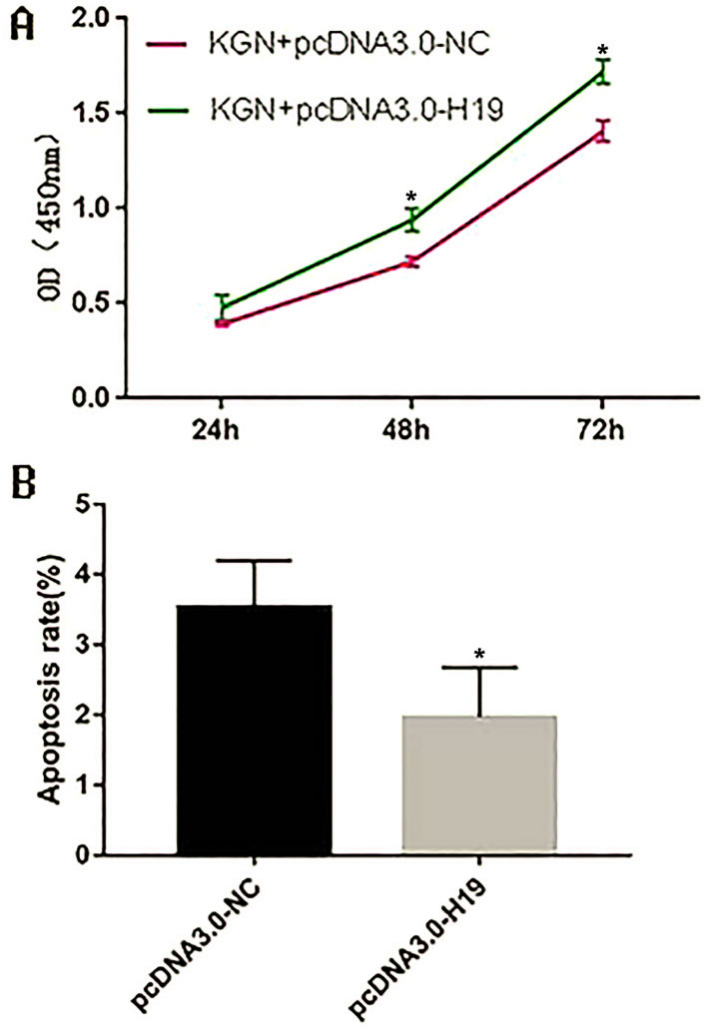
A, Cell proliferation was detected by CCK8 at 24, 48, and 72 h in KGN cells overexpressing *H19*. **B**, Detection of KGN cell apoptosis by flow cytometry after overexpressing *H19*. Data are reported as means±SD. *P<0.05 compared to negative control (NC) (*t*-test).

Compared with the control group, KGN cells in the overexpressed group had enhanced proliferative capacity at 48 and 72 h, and the apoptotic ability was weakened (Figure 3, Supplementary Figure S1). The results indicated that high expression of *H19* had an effect on promoting proliferation and inhibiting apoptosis in KGN cells.

### Silencing lncRNA *H19* in KGN cells inhibited the expression of *CTGF*

**Figure 4:**
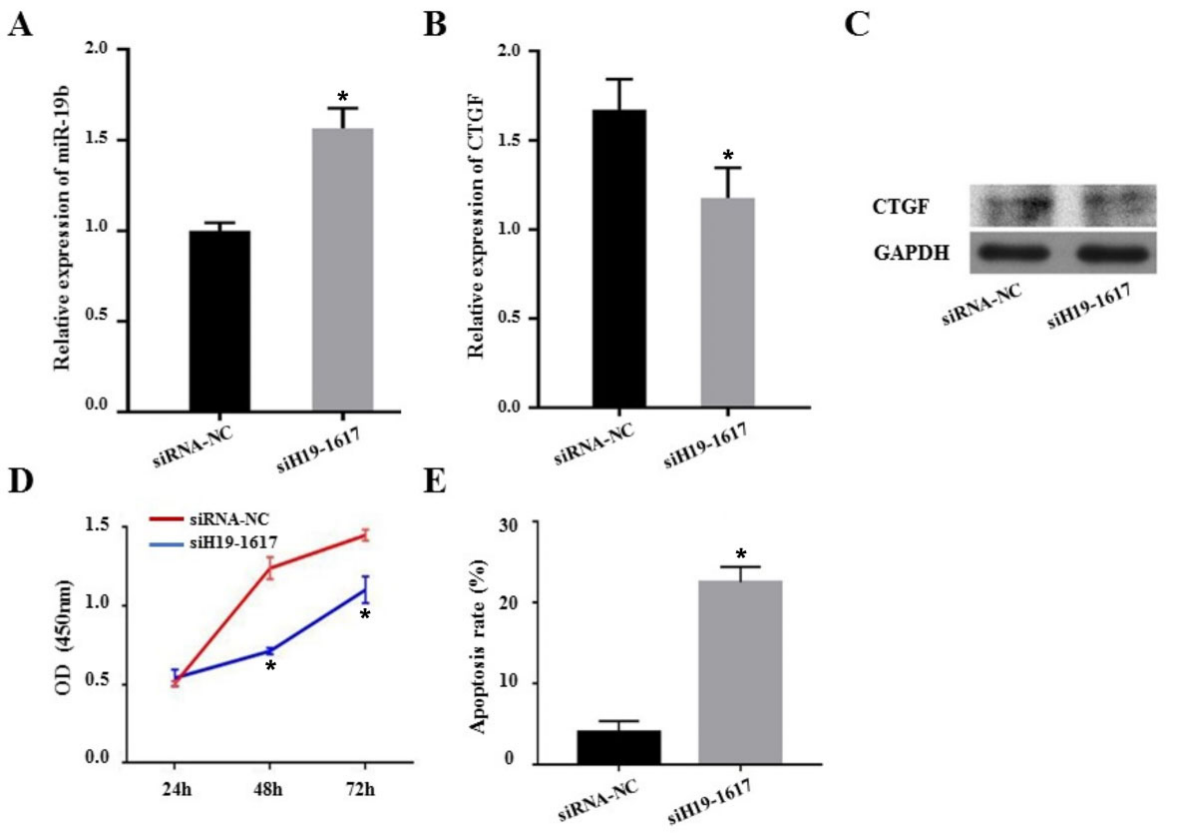
**A**, Expression of miR-*19b* after silencing *H19*. **B**, *CTGF* expression after silencing *H19*. **C**, Expression of CTGF protein after silencing *H19*. **D**, The proliferative ability of KGN cells after silencing *H19* was detected by CCK8 at 24, 48, and 72 h. **E**. KGN cells apoptosis after silencing *H19* was detected by flow cytometry. *P<0.05 compared to negative control (NC). Data are reported as means±SD (*t*-test).

Compared with the corresponding siRNA control group, the expression of miR-*19b* in KGN cells transfected with *siH19-1617* was significantly increased (P<0.05). The *CTGF* reduction of KGN cells transfected with *siH19-1617* was significant (P<0.05) compared to the corresponding siRNA control group. The CTGF protein results were consistent with the results of qRT-PCR. After silencing *H19*, the expression of CTGF protein was decreased (Figure 4A-C).

### Low expression of *H19* inhibited KGN cell proliferation and promoted KGN cell apoptosis

Compared with the siRNA control group, after transfection of *siH19-1617* to KGN cells, the proliferation of KGN cells was weakened at 48 and 72 h, and apoptosis was enhanced (Figure 4D-E, Supplementary Figure S2). The experimental results showed that the low expression of *H19* inhibited the proliferation of KGN cells and promoted the apoptosis of KGN cells.

### Dual luciferase reporter gene

**Figure 5:**
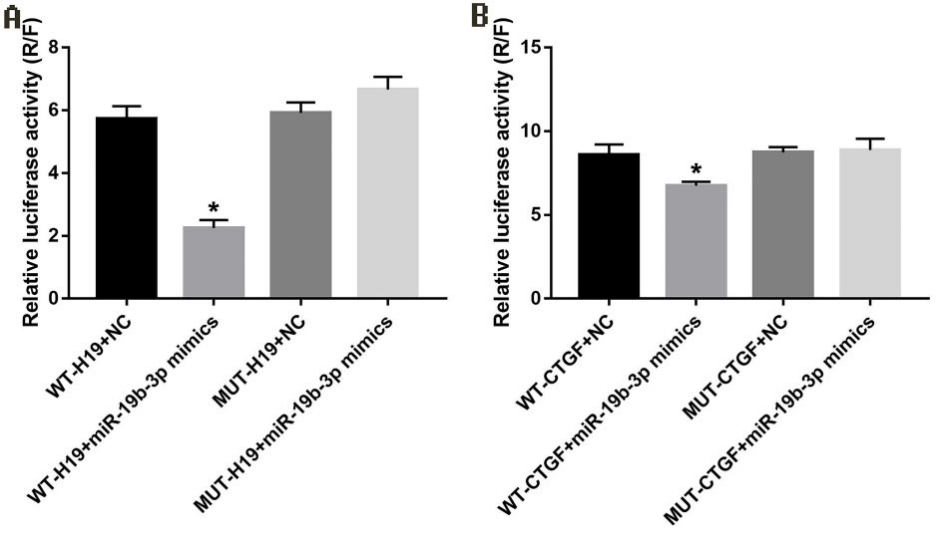
A, lncRNA *H19* and miR-*19b* dual luciferase reporter gene test results (*P<0.05 *vs* WT-H19+NC). **B**, *CTGF* and miR-*19b* dual luciferase reporter gene test results (*P<0.05 *vs* WT-CTGF+NC). Data are reported as means±SD (*t*-test). NC: negative control; WT: wild type; MUT: mutant type.

Compared with the control group, the miR-*19b* mimics co-transfected with wild-type *H19* or 3′ UTR *CTGF* inhibited the fluorescence activity of *H19* and *CTGF* (P<0.05). After mutation of the binding site sequence, miR*-19b* mimics had no effect on the fluorescence activity of the mutant *H19* and *CTGF* (P>0.05). The results of the dual luciferase reporter gene showed that the binding mode between lncRNA *H19* and miR*-19b* and between miR*-19b* and *CTGF* were both directly combined targeted adsorption (Figure 5).

## Discussion

PCOS is a common reproductive endocrine disease in gynecology. Anovulatory infertility caused by PCOS is a serious problem for women of childbearing age (12). PCOS not only affects female fertility but also affects multiple organs in the body (13,14). Currently, there is no effective drug to cure PCOS completely, and many patients suffer from PCOS for life. The pathogenesis of polycystic ovary syndrome is complex, and in-depth study of its specific molecular regulation mechanism can provide a useful target for the diagnosis and treatment of PCOS.

In our previous study, we found that lncRNA *H19* was highly expressed in peripheral blood of patients with PCOS (15). There are many studies on *CTGF* in PCOS that confirm that *CTGF* is highly expressed in PCOS (16), but the reason why is unknown. Is there an upstream regulatory factor of the highly expressed *CTGF*? Is non-coding RNA involved in the regulation of *CTGF* expression? What role does lncRNA *H19* play in the regulation of *CTGF* expression? These issues require in-depth study.

Follicular dysplasia can be observed in patients with PCOS, and granulosa cells around the follicle play a key role in follicular development (17). An important clinical feature of PCOS is infertility caused by abnormal follicular development in women of childbearing age, which is also the main cause of PCOS in patients (17). During growth and development of the oocyte, the oocyte is closely related to the surrounding granulosa cells (18). The nutrients during the growth and development of the oocyte are provided by the surrounding granulosa cells, and the granulosa cells provide growth regulators to promote oocyte maturation (19). The ovaries of PCOS patients contain twice as many follicles at various stages of development. The number of granulosa cells in the ovary of anovulatory PCOS patients is increased compared to the number of granulosa cells in normal ovaries. These conclusions are also confirmed in the PCOS animal model. The relationship between *CTGF* and granulosa cell proliferation phenotype involved in this experiment has been clarified. High expression of *CTGF* can promote granulosa cell proliferation and inhibit granulosa cell apoptosis (20).

The experimental results of our KGN cell study showed that overexpression of *H19* promoted the expression of *CTGF* and inhibited the expression of miR-*19b*. By inhibiting the expression of *H19* by siRNA, the expression of *CTGF* can be inhibited and the expression of miR-*19b* can be promoted. CTGF protein level also confirmed the results of changes in *CTGF* at the RNA level. We studied the effects of lncRNA *H19* on proliferation and apoptosis of KGN cells by CCK8 and flow cytometry. With overexpression of *H19*, KGN cells increased proliferation and decreased apoptosis; with silencing of *H19*, KGN cells decreased proliferation and increased apoptosis. The results showed that lncRNA *H19* can affect the downstream molecular *CTGF* in KGN cells, and abnormally expressed *CTGF* can participate in the pathogenesis of PCOS granulosa cell proliferation and ovarian fibrosis. lncRNA *H19* ultimately affected the pathogenesis of PCOS by regulating the expression of *CTGF*. We found that the direction of regulation of lncRNA *H19* was consistent with that of *CTGF*. With overexpression of lncRNA, *H19* and *CTGF* were also highly expressed. The regulation of lncRNA *H19* was opposite to that of miR-*19b.* After overexpression of lncRNA *H19*, miR-*19b* expression was decreased. After silencing lncRNA *H19*, the situation also satisfied the above. This regulatory trend was consistent with ceRNA. In ceRNA, the regulation trend of lncRNA and mRNA was the same, and the regulation trend of lncRNA and miRNA was the opposite. This provides a basis for our subsequent research on ceRNA mechanisms.

In order to solve the hypothesis that lncRNA *H19* directly binds to *miR*-*19b* via ceRNA mechanism to regulate *CTGF,* we verified by dual luciferase assay that miR-*19b* directly targeted lncRNA *H19* and *CTGF*. Our study showed that lncRNA *H19* can bind to *miR*-*19b*, and the binding ability of *miR*-*19b* and *CTGF* was reduced, which reduced the inhibition of *CTGF* gene expression by *miR*-*19b*, thereby promoting the expression of *CTGF*. In conclusion, lncRNA *H19* affected the expression of *CTGF* by competitively binding *miR*-*19b*, and this abnormally expressed *CTGF* was involved in KGN miR-*19b* cells proliferation.

In this study, we found that was an intermediate molecule in the lncRNA *H19/*miR*-19b/CTGF* regulatory axis. lncRNA *H19* regulated *CTGF* expression via the ‘intermediate bridge' miR-*19b*. miR-*19b* acted as a ‘middleman' in this ceRNA regulatory axis.

This is the first time that the proliferation of granulosa cells from the direction of ceRNA regulation mechanisms was explored. In previous studies, the researchers found that miRNAs can regulate the downstream target gene mRNA, which affects granulosa cell proliferation. In this experiment, we further explored the upstream molecules, finding that lncRNA-miRNA-mRNA regulatory axis regulated the proliferation of granulosa cells.

In summary, this experiment confirmed the role of lncRNA *H19* as a ceRNA in KGN cells, which regulated the expression of *CTGF* through the lncRNA *H19*/miR-*19b*/*CTGF* regulatory axis, and finally played an important role in the pathogenesis of PCOS. However, it should be noted that the ceRNA regulatory network is complex (21,22). Each molecule that can act as a ceRNA (such as lncRNA, circRNA, etc.) contains multiple MREs, which can simultaneously bind to multiple different miRNAs, and the miRNAs ultimately regulate different downstream mRNAs. The regulatory axis has complex biological functions (23). lncRNA *H19* is one of the long non-coding RNAs, which was one of the first discovered lncRNAs. Experiments have been carried out to show the new role of lncRNA *H19* in ceRNA regulatory networks (24), further confirming the complexity and diversity of ceRNA regulatory networks. Therefore, in PCOS, further exploration of lncRNA *H19* and biological phenotypes will help us to understand the pathogenesis of PCOS from a macro perspective.

## Supplementary Material

Click here to view [pdf].

## References

[1] Gateva AT, Velikova TV, Kamenov ZA (2019). Peroxiredoxin 4 levels in patients with PCOS and/or obesity. J Gynecol Obstet Hum Reprod.

[2] He Y, Lu Y, Zhu Q, Wang Y, Lindheim SR, Qi J (2019). Influence of metabolic syndrome on female fertility and in vitro fertilization outcomes in PCOS women. Am J Obstet Gynecol.

[3] Li Y, Xiang Y, Song Y, Wan L, Yu G, Tan L (2019). Dysregulated miR-142, -33b and -423 in granulosa cells target TGFBR1 and SMAD7: a possible role in polycystic ovary syndrome. Mol Hum Reprod.

[4] Wang F, Zhang ZF, He YR, Wu HY, Wei SS (2019). Effects of dipeptidyl peptidase-4 inhibitors on transforming growth factor-beta1 signal transduction pathways in the ovarian fibrosis of polycystic ovary syndrome rats. J Obstet Gynaecol Res.

[5] Gao X, ZY, Li X (2017). Expression of CTGF in polycystic ovary syndrome and its effect on proliferation and apoptosis of ovarian granulosa cells [in Chinese]. J Clin Exp Med.

[6] Liu S, Zhang W, Liu K, Liu Y (2019). LncRNA SNHG16 promotes tumor growth of pancreatic cancer by targeting miR-218-5p. Biomed Pharmacother.

[7] Luo W, Yan D, Song Z, Zhu X, Liu X, Li X (2019). miR-126-3p sensitizes glioblastoma cells to temozolomide by inactivating Wnt/beta-catenin signaling via targeting SOX2. Life Sci.

[8] Chu P, Wang Q, Wang Z, Gao C (2019). Long non-coding RNA highly up-regulated in liver cancer protects tumor necrosis factor-alpha-induced inflammatory injury by down-regulation of microRNA-101 in ATDC5 cells. Int Immunopharmacol.

[9] Karreth FA, Tay Y, Perna D, Ala U, Tan SM, Rust AG (2011). In vivo identification of tumor- suppressive PTEN ceRNAs in an oncogenic BRAF-induced mouse model of melanoma.. Cell.

[10] Huang Z, Lei W, Hu HB, Zhang H, Zhu Y (2018). H19 promotes non-small-cell lung cancer (NSCLC) development through STAT3 signaling via sponging miR-17. J Cell Physiol.

[11] Zhong Z, Li F, Li Y, Qin S, Wen C, Fu Y (2018). Inhibition of microRNA-19b promotes ovarian granulosa cell proliferation by targeting IGF-1 in polycystic ovary syndrome. Mol Med Rep.

[12] Gomez AM, Arteaga S, Ingraham N, Arcara J (2019). Medical conditions, pregnancy perspectives and contraceptive decision-making among young people: an exploratory, qualitative analysis. Contraception.

[13] Hopkins D, Wilson C (2019). Polycystic ovary syndrome in active duty service women: a retrospective analysis. Mil Med.

[14] Schneider D, Gonzalez JR, Yamamoto M, Yang J, Lo JC (2019). The association of polycystic ovary syndrome and gestational hypertensive disorders in a diverse community-based cohort. J Pregnancy.

[15] Qin L, Huang CC, Yan XM, Wang Y, Li ZY, Wei XC (2019). Long non-coding RNA H19 is associated with polycystic ovary syndrome in Chinese women: a preliminary study. Endocr J.

[16] Wang D, Wang W, Liang Q, He X, Xia Y, Shen S (2018). DHEA-induced ovarian hyperfibrosis is mediated by TGF-beta signaling pathway. J Ovarian Res.

[17] Sagvekar P, Kumar P, Mangoli V, Desai S, Mukherjee S (2019). DNA methylome profiling of granulosa cells reveals altered methylation in genes regulating vital ovarian functions in polycystic ovary syndrome. Clin Epigenetics.

[18] Shen M, Li T, Zhang G, Wu P, Chen F, Lou Q (2019). Dynamic expression and functional analysis of circRNA in granulosa cells during follicular development in chicken. BMC Genomics.

[19] Bertoldo MJ, Cheung MY, Sia ZK, Agapiou D, Corley SM, Wilkins MR (2019). Non-canonical cyclic AMP SMAD1/5/8 signalling in human granulosa cells. Mol Cell Endocrinol.

[20] Chang HM, Y Fang, Liu PP, Cheng JC, X Yang, Leung PC (2016). Connective tissue growth factor mediates growth differentiation factor 8-induced increase of lysyl oxidase activity in human granulosa-lutein cells. Mol Cell Endocrinol.

[21] Chen W, Chen X, Wang Y, Liu T, Liang Y, Xiao Y (2019). Construction and analysis of lncRNA-mediated ceRNA network in cervical squamous cell carcinoma by weighted gene co-expression network analysis. Med Sci Monit.

[22] Tu YA, Lin SJ, Chen PL, Chou CH, Huang CC, Ho HN (2019). HSD3B1 gene polymorphism and female pattern hair loss in women with polycystic ovary syndrome. J Formos Med Assoc.

[23] Shao M, Li W (2019). Transcriptional factor regulation network and competitive endogenous RNA (ceRNA) network determining response of esophageal squamous cell carcinomas to neoadjuvant chemoradiotherapy. PeerJ.

[24] Sun W, Lv J, Duan L, Lin R, Li Y, Li S (2019). Long noncoding RNA H19 promotes vascular remodeling by sponging let-7a to upregulate the expression of cyclin D1. Biochem Biophys Res Commun.

